# Effect of Single-Residue Mutations on CTCF Binding to DNA: Insights from Molecular Dynamics Simulations

**DOI:** 10.3390/ijms24076395

**Published:** 2023-03-29

**Authors:** Albert Mao, Carrie Chen, Stephanie Portillo-Ledesma, Tamar Schlick

**Affiliations:** 1 Department of Chemistry, New York University, 100 Washington Square East, Silver Building, New York, NY 10003, USA; azm9134@nyu.edu (A.M.); cc6533@nyu.edu (C.C.); sp5413@nyu.edu (S.P.-L.); 2 Courant Institute of Mathematical Sciences, New York University, 251 Mercer St., New York, NY 10012, USA; 3 New York University-East China Normal University Center for Computational Chemistry, New York University Shanghai, Shanghai 200122, China; 4 Simons Center for Computational Physical Chemistry, New York University, 24 Waverly Place, Silver Building, New York, NY 10003, USA

**Keywords:** CTCF, mutations, molecular dynamics, cancer

## Abstract

In humans and other eukaryotes, DNA is condensed into chromatin fibers that are further wound into chromosomes. This organization allows regulatory elements in the genome, often distant from each other in the linear DNA, to interact and facilitate gene expression through regions known as topologically associating domains (TADs). CCCTC–binding factor (CTCF) is one of the major components of TAD formation and is responsible for recruiting a partner protein, cohesin, to perform loop extrusion and facilitate proper gene expression within TADs. Because single-residue CTCF mutations have been linked to the development of a variety of cancers in humans, we aim to better understand how these mutations affect the CTCF structure and its interaction with DNA. To this end, we compare all-atom molecular dynamics simulations of a wildtype CTCF–DNA complex to those of eight different cancer-linked CTCF mutant sequences. We find that most mutants have lower binding energies compared to the wildtype protein, leading to the formation of less stable complexes. Depending on the type and position of the mutation, this loss of stability can be attributed to major changes in the electrostatic potential, loss of hydrogen bonds between the CTCF and DNA, and/or destabilization of specific zinc fingers. Interestingly, certain mutations in specific fingers can affect the interaction with the DNA of other fingers, explaining why mere single mutations can impair CTCF function. Overall, these results shed mechanistic insights into experimental observations and further underscore CTCF’s importance in the regulation of chromatin architecture and gene expression.

## 1. Introduction

**Figure 1:**
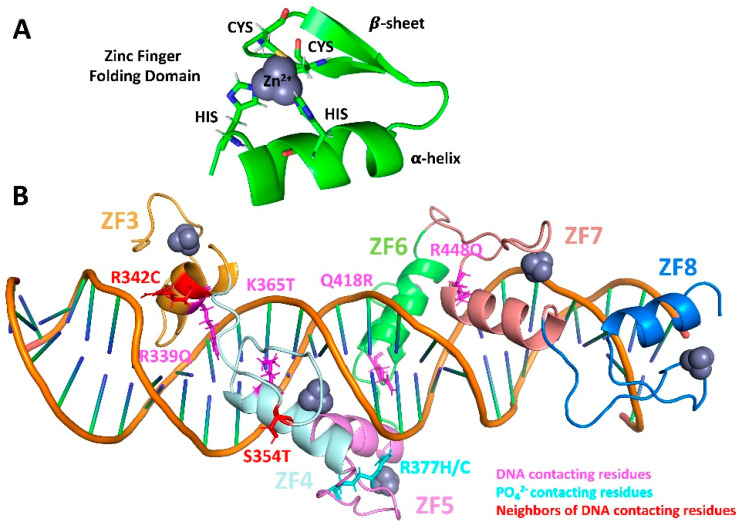
Zinc fingers stabilize the CTCF–DNA structure through protein/DNA contacts. (**A**) Zinc finger folding motif, with the coordination ring and the ββα domain. (**B**) 3D structure of the CTCF–DNA complex showing CTCF’s ZFs 3 to 8 color-coded, the Zn^2+^ ions in violet, the residues that are mutated colored based on their interaction with DNA (pink), DNA PO_4_^2−^ groups (cyan), or with other residues that directly contact the DNA (red). Protein crystal structure obtained from [14].

CTCF is an 11-zinc finger (ZF) protein ubiquitously expressed in eukaryotic cells. It binds to the consensus sequence “CCGCGNGGNGGCAG” at tens of thousands of locations along mammalian genomes [1] using its central domain composed of ten C2H2 class fingers (ZFs 1–10) and one C2HC class finger (ZF 11) [2,3]. Each C2H2 finger unit is formed by a consensus sequence [4], which folds into a ββα domain in the presence of zinc (Figure 1A). In this motif, the zinc is coordinated by two cysteines located near a turn in the antiparallel β sheet and two histidines in the C-terminal portion of the α-helix.

Among the 11 zinc fingers, ZFs 3–7 make direct contact with the DNA and are thus primarily responsible for the association with binding regions [5]. These DNA-contacting fingers are crucial to CTCF’s unique ability to work in tandem with cohesin rings to create topologically associating domains (TADs) through the loop extrusion mechanism [1,5,6,7].

Besides its role in loop extrusion, CTCF acts as an activator, repressor, and insulator; it is also associated with regulation of chromatin architecture, DNA methylation, and tumor suppression [6,8,9]. Thus, the disruption of CTCF–DNA binding by mutations or post-translational modifications often results in the development of various cancers, including endometrial, Wilm’s, and breast cancers [3,5,6,10,11].

Cancer-linked CTCF mutations frequently occur in DNA-contacting residues or adjacent residues within the region of ZFs 3 to 7 [3,5,11]. As opposed to mutations of other DNA binding proteins, CTCF mutations, particularly single-residue mutations, are thought to exhibit a change in function rather than a loss of function, as they affect CTCF binding to some target sites but not to others [3]. CTCF mutations may change the DNA-binding affinity and specificity, as well as the protein orientation [3,5,11,12,13]. While the latter causes cohesin to slip over CTCF, altering TAD size [14], the loss of binding affinity, or the change in binding sites, prevents CTCF from stopping cohesin during loop extrusion at specific sites, thereby elongating or shortening TADs. Thus, changes in TAD size resulting from altered CTCF function can affect normal gene expression [7]. Because CTCF can suppress tumor formation by limiting the expression of oncogenes [12], altered TADs are related to cancer development [13].

While it is clear that CTCF mutations are frequently identified in cancer, the degree to which such mutations alter CTCF’s binding capabilities and structure at the atomic level is not well understood [5,13]. Here, we use molecular dynamics (MD) simulations to further investigate the structural effect of eight different mutations identified in cancer patients [15,16,17,18]: R339Q, R342C, S354T, K365T, R377H/C, Q418R, and R448Q, located in ZFs 3 to 7. Overall, we find that all mutations except R342C and K365T reduce the stability of the DNA–CTCF complex due to the loss of hydrogen bonds or electrostatic interactions. While some mutations destabilize their own zinc finger and directly impact the protein secondary structure and, in turn, protein/DNA interactions, others disrupt the CTCF interactions with the DNA without affecting CTCF structure.

Overall, our atomic-level biophysical findings help better understand the molecular mechanisms involved in the misregulation of gene expression and the development of cancer due to CTCF mutations. They also define foundations for further investigating the impact of these mutations on the chromatin level.

## 2. Results

### 2.1. Single-Residue Mutations Affect Global CTCF Tertiary Structure and Flexibility

**Figure 2:**
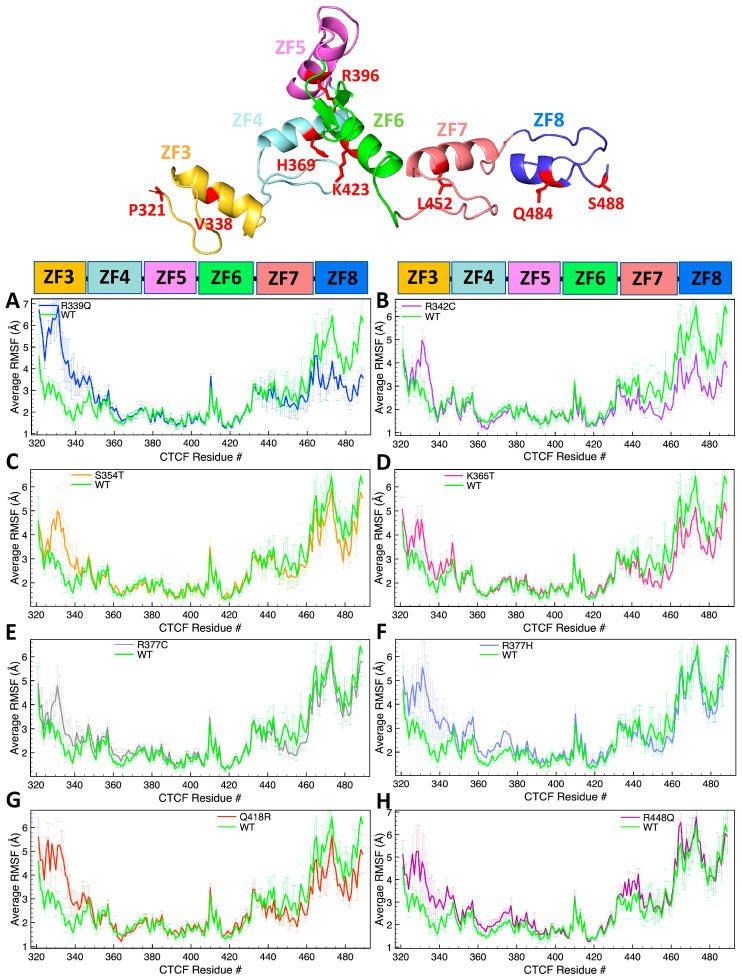
Single-residue mutations alter the average RMSF per residue of CTCF. Panels compare the wildtype system (green curve) to: (**A**) R339Q; (**B**) R342C; (**C**) S354T; (**D**) K365T; (**E**) R377C; (**F**) R377H; (**G**) Q418R; and (**H**) R448Q. The residue position of each zinc finger is shown at the top (ZF3 321–348; ZF4 349–376; ZF5 377–404; ZF6 405–434; ZF7 435–464; and ZF8 465–489). RMSF values are calculated by averaging the RMSF of each of the five replicas. Error bars represent the standard error.

From our 300 ns MD trajectories of wildtype CTCF–DNA and mutant versions based on the crystal complex, as described in Methods, we analyze each system by measuring the root mean squared fluctuation (RMSF) per residue along the 169 CTCF residues (residue 321 to 489) for each of the five replicas and then calculate the average RMSF, as shown in Figure 2. As we show in Supplementary Figure S1, the five replicas have similar RMSFs, indicating that the differences observed for the average RMSF between the WT and mutants likely reflect the mutation rather than trajectory variations (due to the chaotic nature of molecular dynamics [19]).

Mutants R339Q (Figure 2A, blue trace) and R342C (Figure 2B, violet trace), both located on ZF3, increase the RMSF of the region where the mutations occur, indicating that these mutations affect the protein structure. Additionally, in both mutants, the RMSF of ZF8 is reduced compared to the WT system. The pair S354T (Figure 2C, orange trace) and K365T (Figure 2D, magenta trace), both located on ZF4, do not affect the mobility of the region surrounding them. However, like R339Q and R342C, they affect the region of ZF3, although to a lower extent. Mutations of R377 to C (Figure 2E, gray trace) or H (Figure 2F, orchid trace), located between ZFs 4 and 5, increase the RMSF of the surrounding region, with a more pronounced effect in the R377H mutant. Finally, mutation Q418R (Figure 2G, red trace) decreases the mobility of ZF8 and increases the mobility of ZF3, whereas mutation R448Q (Figure 2H, plum trace) increases the mobility of ZF7, where it is located, and the mobility of ZF3.

Thus, some single-residue mutations can affect the flexibility of residues located in the same finger as they occur, such as R339Q, R342C, R377C/H, and R448Q, or, like S354T, K365T, and Q418R, increase the flexibility of other ZFs.

We next study how the mutations affect the global and local structure of CTCF. In particular, we measure the CTCF radius of gyration and the angle between ZFs 7 and 8.

**Figure 3:**
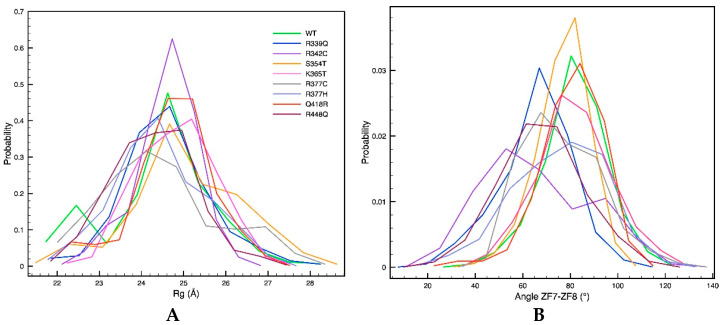
CTCF global and local structural parameters are impacted by single-residue mutations. (**A**) CTCF radius of gyration, and (**B**) angle between ZFs 7 and 8 for the wildtype (WT, green) and mutant proteins calculated using the last 100 ns of five trajectories per system.

Figure 3A shows the probability distribution for the radius of gyration of the wildtype and mutant proteins. The wildtype protein has a narrow distribution with a well-defined peak at around 24.5 Å, and a smaller peak at 22.5 Å that corresponds to one replica out of the 5 that is less extended and more globular compared to the others. All mutants show less-defined probability distributions with shifted or wider peaks. Mutants R339Q, S354T, K365T, R377C, and R448Q show the largest differences compared to the wildtype protein, with wider distributions and peaks shifted to smaller or larger values. On the other hand, the mutants R342C, R377H, and Q418R show distributions that resemble the one of the wildtype protein, with peaks at similar values.

Similarly, as shown in Figure 3B, most mutants exhibit ZF7–ZF8 angle probability distributions that are less defined, and in some cases, such as for R339Q, R342C, R377C, and R448Q, shifted to smaller values with respect to the wildtype protein. Because this angle is important for CTCF binding to the DNA in a sequence non-specific manner [14], its alteration could affect CTCF normal binding.

**Figure 4:**
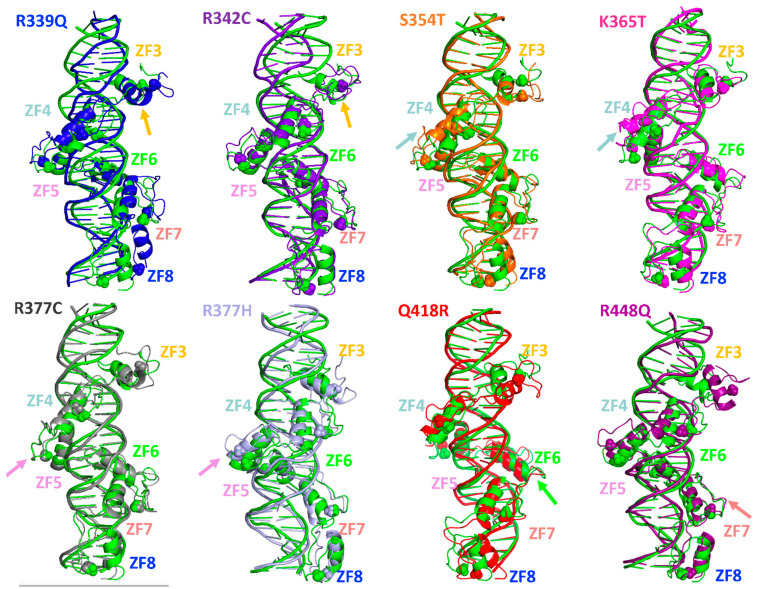
CTCF secondary and tertiary structure is impacted by single-residue mutations. Shown for each mutant is the representative structure of the most populated cluster among its five independent trajectories. Alignment is based on the wildtype protein (green) and each mutant, with arrows indicating the position of each mutation.

Overall, as shown in Figure 4 for the alignment between the representative structure of the most populated cluster of the wildtype protein and each mutant, the effect of all mutations on the CTCF global secondary and tertiary structure and on the ZF7–ZF8 angle is moderate. However, the altered sequences could affect the binding affinity of CTCF and thus the stability of the complex. In the following subsection, we analyze how the CTCF–DNA complex is impacted by each mutation.

### 2.2. Single-Residue Mutations Affect the CTCF–DNA Complex Stability

To determine to what extent single-residue mutations affect the binding of CTCF to the DNA and, thus, complex stability, we calculate the binding energy using the MM- GBSA method, as implemented in AMBER20 (see Methods). As reported in Table 1, all mutant complexes except for R342C and K365T show lower binding energy compared to the wildtype protein, possibly destabilizing. Although the values are all within the same range of error, the comparison of many similar systems can be indicative of trends. The large error bars probably result from the one-trajectory approximation used to calculate the binding energies in which the structures and energies of the isolated CTCF and DNA molecules are obtained from the trajectories of the CTCF–DNA complexes.

This loss of stability can be a product of several factors. For example, mutations may disrupt specific stabilizing hydrogen bonds between the CTCF and DNA. As reported in Table 2, all mutants except for R342C and Q418R exhibit an overall loss of hydrogen bonds compared to the wildtype protein.

**Figure 5:**
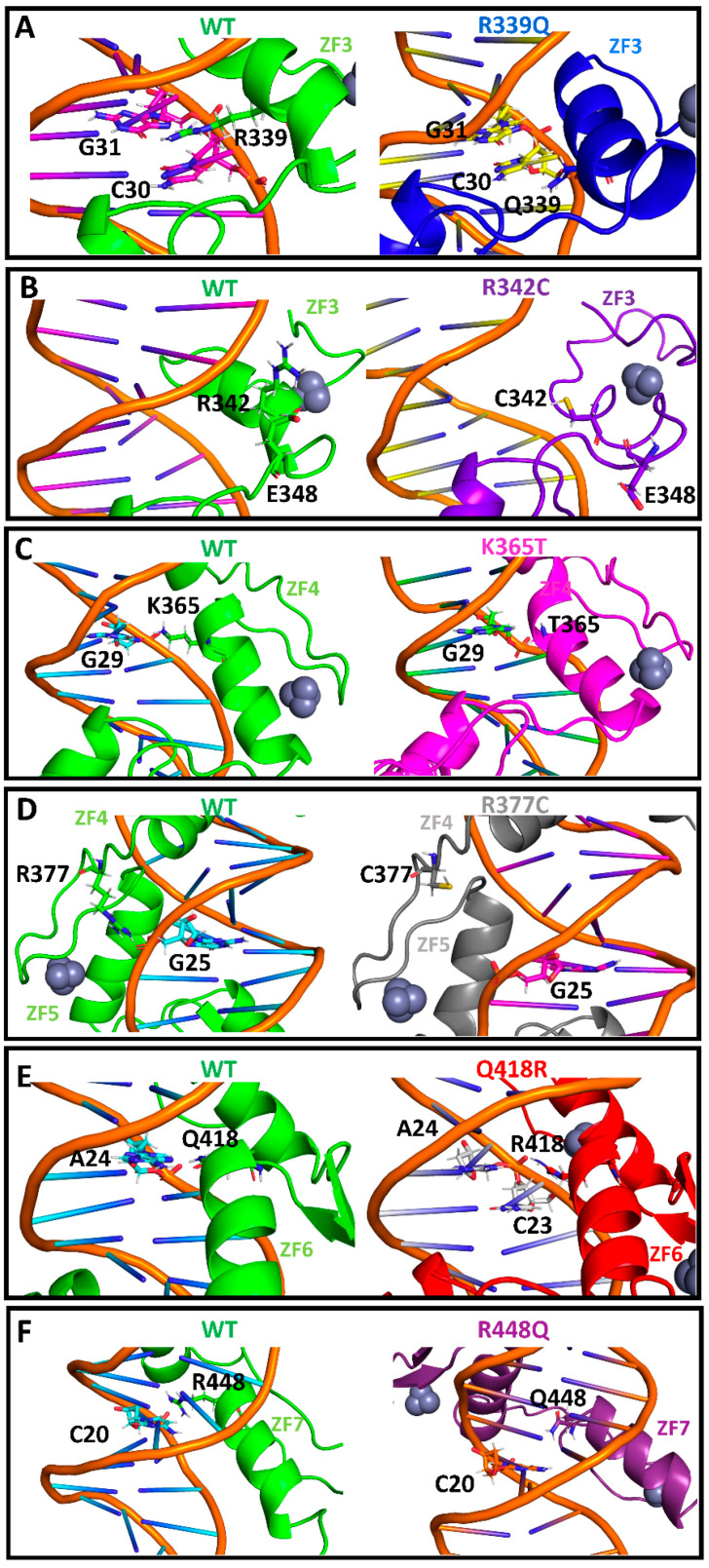
Single-residue mutations affect hydrogen bonding networks between CTCF and DNA. Shown is the 3D structure of the most representative cluster for the WT (all panels, green), R399Q ((**A**), blue), R342C ((**B**), violet), K365T ((**C**), magenta), R377C ((**D**), gray), Q418R ((**E**), red), and R448Q ((**F**), plum) proteins, with specific hydrogen bond interactions.

**Figure 6:**
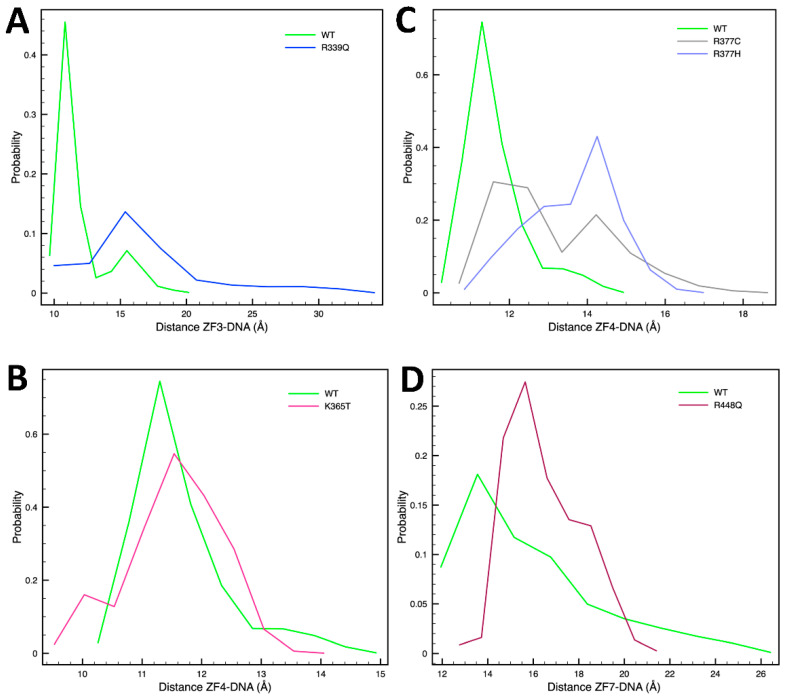
Single-residue mutations affect the interaction between the α-helix of specific ZFs and the DNA. Shown is the probability distribution for the distance between the center of mass of a ZF’s α-helix and the DNA for: (**A**) ZF3 of WT vs. R339Q, (**B**) ZF4 of WT vs. R337C/H, (**C**) ZF4 of WT vs. K365T, and (**D**) ZF7 of WT vs. R448Q. Distributions are calculated using the last 100 ns of each of the five trajectories per system.

Residue R339, located at a DNA-binding position in ZF3, interacts via two hydrogen bonds with a guanine (G31) and cytosine (C30) in the wildtype protein (Figure 5A). These interactions are lost upon its mutation to Q (Figure 5A); as shown in Table 2, all three hydrogen bonds established between the ZF3 and the DNA are lost in this mutant. Indeed, ZF3 is shifted away from the DNA, as shown by the increase in the distance between the center of mass of its α helix and the DNA region composed of residues G28, G29, C30, and G31 (Figure 6A). R342, also located in ZF3, does not directly interact with the DNA. However, this residue establishes a hydrogen bond with the residue E348 that stabilizes the ZF3′s α-helix (Figure 5B). When this interaction is lost upon its mutation to C, ZF3 is destabilized, and the finger’s α-helix partially unfolds (Figure 5B and Supplementary Table S1). However, this does not affect the hydrogen bonds with the DNA (Table 2). Indeed, there is an increase in the total number of hydrogen bonds compared to the WT system, which agrees with the higher binding energy obtained for this system. Thus, the mechanism of action of this mutant might not be related to the formation of a less stable complex but to the presence of a residue sensitive to oxidation, which could affect CTCF function. The only cysteine residues of wildtype CTCF are coordinating the zinc ion and cannot be oxidized. However, introducing this cysteine residue could induce the oxidation of CTCF, and this could affect its function.

Residue S354 is located in ZF4 and is one of the two X residues in the motif “CXXC”, where the two cysteines coordinate the Zn^2+^. As can be seen in Table 2, all ZFs except for ZFs 6 and 7 lose hydrogen bonds upon the S354T mutation, with ZF4 being the most affected. Indeed, ZF4 is shifted away from the DNA (Supplementary Figure S2), explaining the loss of hydrogen bonds. K365, also located in ZF4, directly interacts with the DNA through a hydrogen bond with G29 (Figure 5C). When K365 is mutated to T, the hydrogen bond is lost, but the structure of the ZF’s α-helix is maintained (Figure 5C). The distance between the center of mass of the ZF’s α-helix and the DNA segment containing the residues G25, A26, G27, G28, and G29 is similar to the wildtype system (Figure 6B), although smaller distances are visited during the simulation, indicating a possible closer interaction. This could explain the higher binding energy compared to the wildtype system.

Residue R377 is located in the link region between ZF4 and ZF5 and belongs to the consensus region “TGEK(R)P”, where the K/R in the fourth position usually interacts with the DNA [4]. As shown in Figure 5D, R377 interacts through hydrogen bonds with the PO_4_^2−^ group of G25. When it is mutated to C (Figure 5D) or H (Supplementary Figure S3), the link region between ZF4 and ZF5 is shifted away from the DNA. Because this region is important for the correct positioning of the ZFs, these mutations affect the interaction of ZF4 with the DNA, reflected by the total loss of hydrogen bonds of this finger (Table 2) and by the increase in the distance between the center of mass of ZF4′s α-helix and the DNA region containing the residues G25, A26, G27, G28, and G29 (Figure 6C) compared to the wildtype system. The effect is more pronounced in the R377H mutant.

Residue Q418 is located on ZF6 and interacts through hydrogen bonds with residue A24, located at the center of the palindromic region “CCACCAGGTGG”. A24 is thought to be important for the correct directional CTCF binding [14]. In the wildtype protein, Q418 interacts exclusively with A24 (Figure 5E) through a hydrogen bond with a frequency of 63% (i.e., within 63% of the analyzed trajectories). However, upon its mutation to R, the residue mostly interacts with C23 (Figure 5E), forming a hydrogen bond (for 47% of the time) and reducing the occurrence of the hydrogen bond with A24 to 27%. Such a change could affect the correct directional binding of CTCF to the DNA, causing cohesin to slip over CTCF, altering the loop extrusion and thus the TAD size [14].

Finally, residue R448 is located on ZF7 and interacts with C20, a highly conserved nucleotide (Figure 5F). This interaction positions the ZF’s α-helix close to the DNA. Upon its mutation to Q, this hydrogen bond is lost (Figure 5F and Table 2), and the ZF’s α-helix shifts away from the DNA (Figure 6D).

**Figure 7:**
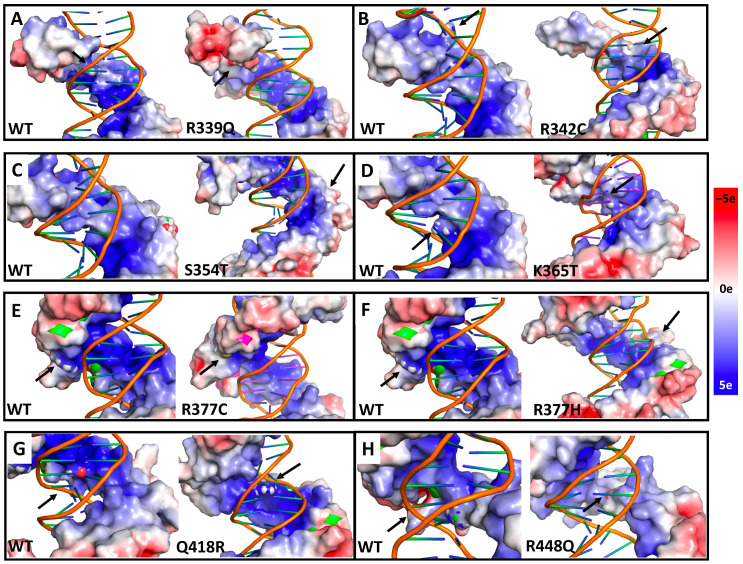
Single-residue mutations affect the electrostatic potential of CTCF. 3D structure of the most representative cluster for the WT (all panels), R399Q (**A**), R342C (**B**), S354T (**C**), K365T (**D**), R377C (**E**), R377H (**F**), Q418R (**G**), and R448Q (**H**) proteins, showing the electrostatic potential on the area surrounding the mutation. Color scale goes from 5e (blue) to 0e (white) to −5e (red) (from basic to acidic). Arrows indicate the location of the mutation.

The loss of stability in the mutant CTCF–DNA complexes can also be explained through changes in the electrostatic potential of CTCF upon the introduction of mutations, especially when a basic residue is replaced by a neutral amino acid. Figure 7 shows how the electrostatic potential of the CTCF wildtype is impacted, with arrows pointing to the mutation position.

The R339Q complex exhibits an important change in the electrostatic potential, where it becomes almost neutral (change from blue to white) (Figure 7A). R342C also alters the electrostatic potential, but the effect is less pronounced (Figure 7B).

S354 is located on the surface region of CTCF and does not interact with DNA. As expected, due to the similar nature, its mutation to T does not alter the electrostatic potential (Figure 7C). The mutation of K365 to T shows electrostatic potential values that are slightly less positive than those of the wildtype system (Figure 7D).

The mutations of R377 to C or H make the electrostatic potential slightly less positive (Figure 7E,F). However, as this residue does not directly interact with the DNA, the impact on the complex stability may be not as much as in the case of R339Q.

When Q418 is mutated to R, the electrostatic potential becomes more positive (Figure 7G). This change could increase the interactions between CTCF ZF6 and the DNA. However, because a specific interaction between Q418 and A24 is needed for the correct positioning of CTCF, this change could imply impaired CTCF function.

Finally, the mutation of R448 to Q significantly shifts the electrostatic potential to more negative values (change from blue to white) (Figure 7H), similar to R339Q (Figure 7A). Thus, mutations of R to Q are highly disruptive, probably due to the large size of the Q residue.

## 3. Discussion

We have investigated the atomic-level effects of common CTCF mutations associated with human cancers by molecular dynamics simulations. Overall, all mutations except for R342C and K365T appear destabilizing for the CTCF–DNA complex, but the mechanism of action is mutation-dependent.

The mutant R339Q produces an increase in the flexibility of ZFs 3 and 4, a decrease in the radius of gyration, and a decrease in the angle ZF7–ZF8. At the local level, the interaction of R339 with the DNA, through hydrogen bonds with the residues C30 and G31 and via electrostatic interactions, is lost upon the mutation, displacing the ZF3′s α-helix. Because the binding of ZF3 to the DNA is important for the correct directional binding of CTCF [14], this effect could impair CTCF normal directional binding and, thus, its interaction with cohesin.

The changes we observe agree with experiments showing that the R339Q mutant diminishes DNA binding, impairs the CTCF tumor suppressor activity, and affects its regulatory transcriptional activity [20].

The mutant R342C exhibits higher binding energy and more hydrogen bonds than the wildtype system, indicating that it does not destabilize the CTCF–DNA interaction. However, it slightly alters the ZF3′s secondary structure, which could affect the finger interaction with DNA or the interaction of CTCF with its partner cohesin. Additionally, the introduction of a cysteine residue that is prone to oxidation could impact CTCF function. This mutant has been detected in individuals with neurodevelopmental disorders, and it was related to a broad deregulation of genes [21]. In one study, the authors predict that the disruption of the hydrogen bond between R342 and E348 would destabilize the ZF3 structure [21]. Our results confirm this destabilization of ZF3, thus providing a rationale for the correlation between this mutation and the development of cancer and other diseases.

The mutant S354T is located on the surface of the protein and does not interact with DNA. It does not significantly affect the global properties of CTCF, but it affects the total number of hydrogen bonds, with ZF4 losing most hydrogen bonds and shifting away from the DNA. This mutation appears to act by affecting the interaction between ZFs 3 to 5 and DNA. Additionally, because of its location, it could potentially affect the interaction between CTCF and other binding partners involved in its insulator function [22].

Mutant K365T shows a radius of gyration and a ZF7–ZF8 angle similar to those of the wildtype protein and a net loss of only one hydrogen bond. Thus, this mutation does not seem to affect the interaction between ZF4, where it is located, and the DNA. Additionally, it shows a higher binding energy than the wildtype protein. Although a similar mutant, K365A, exhibits a decrease in the DNA binding affinity [14], that is not the case for K365T. That ZF4 is located closer to the DNA in the K365T mutant could be related to experimental observations showing a pro-survival gain of function for this mutant that contributes to tumorigenesis [12]. Thus, the shifting of ZF4 towards the DNA that generates new hydrogen bond interactions could in turn trigger the protein to bind to new DNA sequences and thus lead to a gain of function.

The mutations of R377 to H or C disrupt the link region between the ZFs 4 and 5 and the ZF4 itself, which shifts away from the DNA. These results correlate with experiments showing a complete loss of tumor suppressive effects, an affected transcriptional regulation activity, and a diminished DNA binding of CTCF upon the R377H and R377C mutations [12,20,23].

The mutant Q418R in ZF6 does not significantly affect the flexibility or global geometry of CTCF. However, while Q418 interacts exclusively with the A24 residue located in the center of the palindromic region of the CTCF binding site, R418 additionally interacts with the residue C23. CTCF-binding sites are frequently mutated in cancer, and the mutations are predominately located at the A24 residue [24]. This suggests that the interaction between A24 and Q418 is essential for the correct directional binding of CTCF, and thus, the change in hydrogen bonds with Q418R mutation likely affects CTCF’s binding to the DNA.

Finally, the mutant R448Q is disruptive for the ZF7, where it is located, increasing its flexibility, shifting it away from the DNA, and altering the electrostatic potential. These effects suggest that this mutation will be disruptive to CTCF’s function, in agreement with experimental results showing that R448Q does not bind to growth-regulatory genes such as MYC, PIM1, p19ARF, and Igf2/H19 [3]. Additionally, that the hydrogen bond with nucleotide C20 is lost upon this mutation agrees with experiments showing that the methylation of this nucleotide blocks CTCF’s capability to form chromatin loops and to facilitate enhancer/promoter interactions [25].

Although our results are obtained by relatively short MD trajectories, the comparison among many similar complexes, each with multiple replicas, is meaningful. Our modeling is also based by necessity (due to unavailability of structural information) on a truncated CTCF–DNA crystal complex containing ZF’s 3 to 8, thus not immediately applicable to the whole CTCF–DNA complex or in vivo experiments. Yet, clearly, our results underscore the potential effect of single-residue mutations in DNA-binding proteins and the associated DNA/protein complexes. Such disruptions of hydrogen binding, electrostatic interactions, and zinc finger stabilization by single-residue mutations of CTCF provide atomic-level insights into the molecular mechanisms that can explain the relationship between CTCF mutations and the development of human cancers. They further provide the groundwork for modeling these mutations in the context of longer CTCF–DNA complexes and chromatin fibers, a natural future goal [26].

## 4. Materials and Methods

### 4.1. Initial Systems Models

The crystal structure of the wildtype (WT) CTCF–DNA complex containing ZFs 3 to 8 and a DNA chain of 27 nucleotides was obtained from the Protein Data Bank (PDB code: 5YEF) [14]. Models were created for each mutant by altering appropriate residues of the WT structure. In particular, the atoms of the side chain of the residue to be mutated were deleted, and the residue name was changed according to the desired mutation. The Tleap module of Amber was used to construct the missing side chain atoms using the residue templates of Amber in which most side chain dihedrals are predicted to be in trans/trans conformations. All residues were protonated considering physiological pH = 7.4, except for the cysteines and histidines that coordinate the Zn^2+^, which need to be deprotonated to establish the coordination [27,28]. Figure 1B shows the locations of mutated residues in color-coding by their interaction with DNA nucleobases, DNA PO_4_^2−^ groups, or other residues that contact DNA.

### 4.2. Molecular Dynamics Simulations

Molecular Dynamics (MD) simulations were performed using the AMBER20 software package [29,30,31]. Force field parameters for the deprotonated histidines coordinating the Zn^2+^ were obtained from [32], and Zn^2+^ ions coordinated by four residues (two histidines and two cysteines) were modeled through the dummy atom approach [32]. In this approach, four identical dummy atoms (DZ) are tetrahedrally attached to the zinc ion (ZN), and the atomic charge of the zinc is evenly transferred to the four dummy atoms that interact with other atoms electrostatically. In this manner, the dummy atoms mimic the zinc’s 4s4p3 vacant orbitals that accommodate the lone-pair electrons of zinc coordinates, simulating zinc’s propensity for the four-ligand coordination. See the force field parameters for the tetrahedral zinc ion in Table 3. The Ff19sb and parmbsc1 force fields were used for the protein and DNA, respectively [33,34]. Chloride ions were added to neutralize the complex.

Each system was then solvated using the OPC water model [35] in rectangular boxes with a minimum distance between the solute and the box edge set to 10 Å. Sodium and chloride ions were added based on the water box volume to ensure a 150 mM salt concentration.

The solvated systems were then energetically minimized using a force constant of 500 kcal/mol Å^2^ to restrain the CTCF–DNA complex. Five thousand iterations were performed using the steepest descent algorithm (SD), followed by 5000 iterations with the conjugated gradient algorithm (CG). This was followed by a second minimization of the same number of iterations but without restraints. The minimized systems were then heated from 0 to 300 K (room temperature) over 0.1 ns with a 2 fs time step using the Langevin Thermostat with a collision frequency of 2 ps^−1^ for temperature control. The systems’ density was then equilibrated to 1 g/cm^3^ over 1 ns using the Berendsen barostat with a pressure relaxation time of 1 ps combined with the Langevin thermostat for temperature control (300 K). Finally, 300 ns production MDs were performed (with frames saved every 1000 steps) under constant temperature and volume for five independent replicas of the WT and each of the mutant systems. Random seed numbers were generated by the Langevin algorithm to account for variability. SHAKE was enabled for all bonds involving hydrogen [36], and an 8 Å nonbonded cutoff was set for nonbonded interactions treated with the Particle Mesh Ewald (PME) algorithm [37]. Convergence was monitored by calculating the root mean square deviation of the CTCF–DNA complex atoms, using, as a reference, the final structure of the density equilibration simulation (Supplementary Figure S4). The last 100 ns of each of the 5 replicas were used for analysis.

We restricted the length of the trajectories to 300 ns because, within the longer *µ*s simulations, some of the systems became unstable, likely due to the truncated CTCF–DNA complex modeled based on the available crystal structure. Currently, no crystal structure of a CTCF–DNA complex containing all 11 ZFs is available. Additionally, the structure of the disordered N- and C-terminal regions flanking the DNA binding region of 11 ZFs is unknown. Modeling the complete CTCF structure is not straightforward without an experimental reference, explaining our practical approach here.

### 4.3. Binding Free Energy Calculations

Binding free energy calculations of the CTCF–DNA complexes were performed using the Python version of the Molecular Mechanics Generalized Born Surface Area (MM–GBSA) method, as implemented in AMBER20 [38]. In particular, we use the single-trajectory approximation in which the structures of the DNA, CTCF, and CTCF–DNA complex are extracted from the CTCF–DNA complex trajectories. We calculate the binding energy for each of the five independent trajectories for each system using the last 100 ns of each trajectory and extracting frames every 0.02 ns to determine average and standard deviations.

The binding free energy is calculated as:

∆*G_bind_* = *G_Complex_* − *G_CTCF_* − *G_DNA_*, (1)

where *G_complex_*, *G_CTCF_*, and *G_DNA_* are the free energies of the complex, CTCF, and DNA, respectively. These free energies are computed by MMGBSA as follows:

∆*G_Complex,CTCF,DNA_* = *E_MM_* − *Gsolv* − *TS*, (2)

where *T* is the temperature, *S* the entropy, and

*E_MM_* = *E_ele_* + *E_vdw_* + *E_int_*, (3)

and

∆*E_solv_* = *G_gb_* + *G_np_*. (4)

*E_ele_*, *E_vdw_*, and *E_int_* are the electrostatic, van der Waals, and internal energy, respectively. ∆*E_solv_* is the solvation free energy, which is decomposed in the electrostatic solvation energy (*G_gb_*), and the non-electrostatic solvation energy (*G_np_*). The value *G_gb_* is calculated with the Generalized Boltzmann solvation model using dielectric constants of 1 and 80 for the solute and solvent, a salt concentration of 150 mM, and the set of effective atomic Born radii developed in [39]. We estimate *G_np_* as:

*G_np_* = *γ* × *SASA* + *β*, (5)

where *γ* and *β* are two empirical constants set to 0.00542 kcal mol^−1^ Å^−2^ and 0.92 kcal mol^−1^, and SASA is the solvent accessible surface area determined using a probe radius of 1.4.

For simplicity, we neglect the entropic contribution. Entropy can be neglected if states of similar entropy are compared, as in the case of DNA binding to mutant CTCFs which vary only by one residue with respect to the wildtype system. Additionally, the normal mode analysis calculations are expensive and have large errors that could produce binding free energies with significant errors.

### 4.4. Structural Analysis Calculations

All analyses were performed using the CPPTRAJ module [40] of AMBER20. The mass-weighted radius of gyration (*R_g_*) was analyzed to assess the compactness and overall shape of the CTCF protein. *R_g_* is calculated as:

(6) $$Rg=\sqrt{I/M},$$

where *I* is the moment of inertia about any axis of rotation, and *M* is the mass of the protein. The assumption that the mass of the protein is concentrated at a distance *r* from the axis of rotation gives a new form of Equation (6):

(7) $$R_{g}=\sqrt{\sum mr^{2}/M}.$$

The fluctuation of residues in the CTCF–DNA complexes is determined from the root mean squared fluctuation (RMSF). The RMSF for a given atom *i* is calculated as:

(8) $$RMSFi=\sqrt{{\langle \left(xi-\langle xi\rangle \right)\rangle }^{2}},$$

where *x* denotes atomic positions, and the averages are over all input frames.

We calculate the RMSF for each of the five trajectories per system and then calculate the average RMSF for each residue and the standard error determined as the standard deviation by the sample size’s square root.

The CTCF secondary structure was measured using the DSSP Kabsch and Sander algorithm [41] that assigns secondary structure elements such as α helices, β sheets, and loops based on hydrogen-bonded and geometrical features.

Clustering analysis of the structures along the trajectories was performed using the K-means algorithm, selecting the number of clusters as 10. To select the optimal number of clusters used to partition the ensemble, we performed clustering for the WT system using 10, 15, 20, and 25 clusters and determined the population and silhouette value for each cluster (Supplementary Figure S5). An average silhouette value for each cluster, ranging from −1 to 1, implies good separation from neighboring clusters, for positive higher values [42]. We choose 10 clusters because this value produces an exponential decrease in the clusters population and the highest average silhouette value. The most representative cluster was used for the visualization of the CTCF–DNA structure and the calculation of the electrostatic potential. See Supplementary Table S2 for cluster populations and Supplementary Figure S6 for the superposition between the representative structures of clusters 1 and 2, the most populated.

Hydrogen bonds between the CTCF and DNA were calculated using geometric and frequency criteria. Namely, we use a cutoff of 135° for the D-H…A angle, a cutoff of 3.0 Å for the D…A distance, and a criterion of 20% for the frequency of hydrogen bond presence in the simulation.

Finally, we measure the angle formed between ZFs 7 and 8 as the angle between the two vectors defined from the N- to the C-terminal region of the α helices of ZFs 7 and 8 (Supplementary Figure S7). Molecular structures were visualized with Pymol [43].

## Figures and Tables

**Table 1:** Average binding energy in kcal/mol calculated for each CTCF–DNA complex over the last 100 ns of simulation for each of the five trajectories per system.

WT	R339Q	R342C	S354T	K365T	R377H	R377C	Q418R	R448Q
−73 ± 11	−53 ± 5	−81 ± 10	−72 ± 13	−82 ± 16	−65 ± 12	−69 ± 10	−69 ± 7	−61 ± 15

**Table 2:** Number of hydrogen bonds in each ZF and within the complex are sensitive to CTCF mutations in CTCF–DNA complexes. Values are measured over the last 100 ns of the trajectory for each of the five trajectories per system.

ZF	WT	R339Q	R342C	S354T	K365T	R377H	R377C	Q418R	R448Q
		(ZF3)	(ZF3)	(ZF4)	(ZF4)	(ZF4–5)	(ZF4–5)	(ZF6)	(ZF7)
3	3	0	4	1	3	2	3	4	0
4	6	2	4	1	4	0	0	3	0
5	4	4	3	2	3	3	3	4	3
6	4	6	8	6	5	6	5	7	5
7	1	2	4	2	2	1	4	0	0
Total	18	14	23	12	17	12	15	18	8

**Table 3:** Bonded force field parameters of the tetrahedral zinc ion coordinated by four dummy atoms.

Bond	k (kcal/mol Å^2^)	Req (Å)	
DZ-ZN	540	0.90	
DZ-DZ	540	1.47	
**Angle**	**k (kcal/mol radian** **^2^)**	**Teq (deg.)**	
DZ-ZN-DZ	55	109.50	
DZ-DZ-DZ	55	60.0	
DZ-DZ-ZN	55	35.25	
**Dihedral**	**IDIVF**	**Vn/2 (kcal/mol)**	***γ* (deg.) N**
ZN-DZ-DZ-DZ	1	0	35.3
DZ-ZN-DZ-DZ	1	0	120.0
DZ-DZ-DZ-DZ	1	0	70.5

## Data Availability

The raw data and structures of the two most populated clusters have been submitted to the public repository Zenodo under: https://doi.org/10.5281/zenodo.7733730.

## Supplementary Materials

The supporting information can be downloaded at: https://www.mdpi.com/article/10.3390/ijms24076395/s1.

## Abbreviations

The following abbreviations are used in this manuscript:

CG	Conjugated Gradient
CTCF	CCCTC-binding factor
MD	Molecular Dynamics
MM-GBSA	Molecular Mechanics Generalized Born Surface Area
Rg	Radius of gyration
RMSD	Root Mean Squared Deviation
RMSF	Root Mean Squared Fluctuation
SD	Steepest Descent
TAD	Topologically Associating Domain
WT	Wildtype
ZF	Zinc Finger
