# Supplementary material for: Effect of Single-Residue Mutations on CTCF Binding to DNA: Insights from Molecular Dynamics Simulations
Source: Int J Mol Sci. 2023 Mar 29;24(7):6395. doi: 10.3390/ijms24076395 (PMC10094706; doi:10.3390/ijms24076395)
Supplement: Supplementary file 1 [file ijms-24-06395-s001.zip › ijms-2184529-supplementary.pdf]

Supporting Information

Effect of Single-Residue Mutations on CTCF Binding to DNA: Insights from Molecular Dynamics Simulations

Albert Mao [1,†], Carrie Chen [1,†], Stephanie Portillo-Ledesma [1] and Tamar Schlick [1,2,3,4,*]

[1] Department of Chemistry, New York University, 100 Washington Square East, Silver Building, New York, NY 10003, USA
[2] Courant Institute of Mathematical Sciences, New York University, 251 Mercer St., New York, NY 10012, USA
[3] New York University-East China Normal University Center for Computational Chemistry, New York University Shanghai, Shanghai 200122, China
[4] Simons Center for Computational Physical Chemistry, New York University, 24 Waverly Place, Silver Building, New York, NY 10003, USA
* Correspondence: schlick@nyu.edu
† These authors contributed equally to this work.

1) Supporting Figures

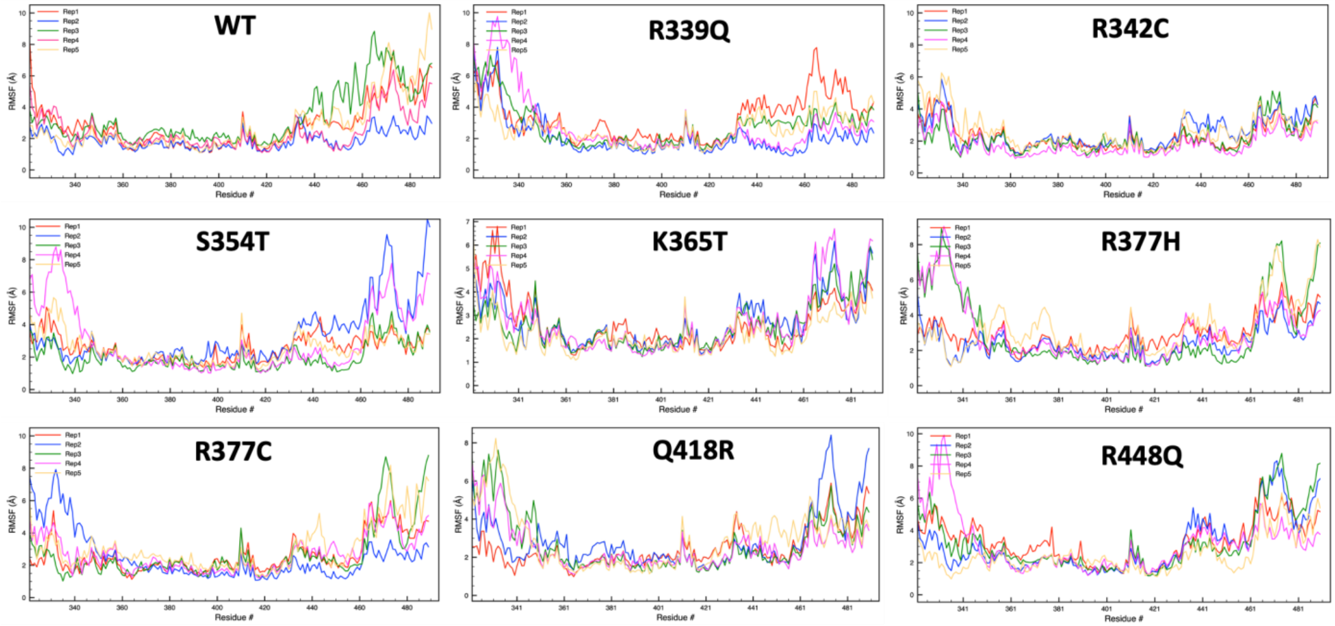

Supplementary Figure S1. Root mean squared fluctuation calculated using the last 100 ns of simulation of each of the five replicas of the wildtype and mutant proteins.

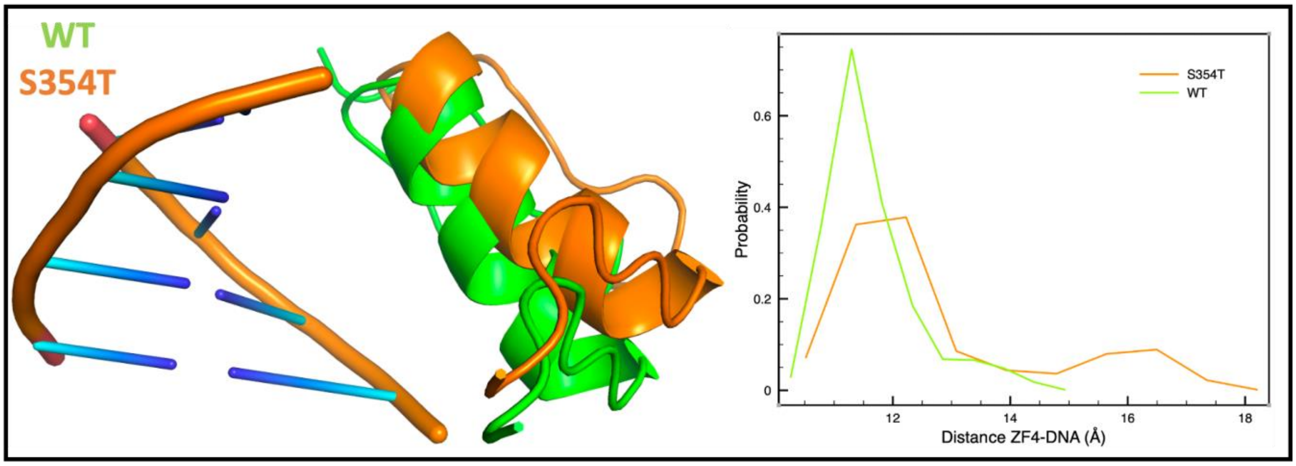

Supplementary Figure S2. Left: overlapping between WT and S354T protein showing the movement of ZF4. Right: Probability distribution for the distance between ZF4 and DNA segment containing nucleotides G25, A26, G27, G28, and G29.

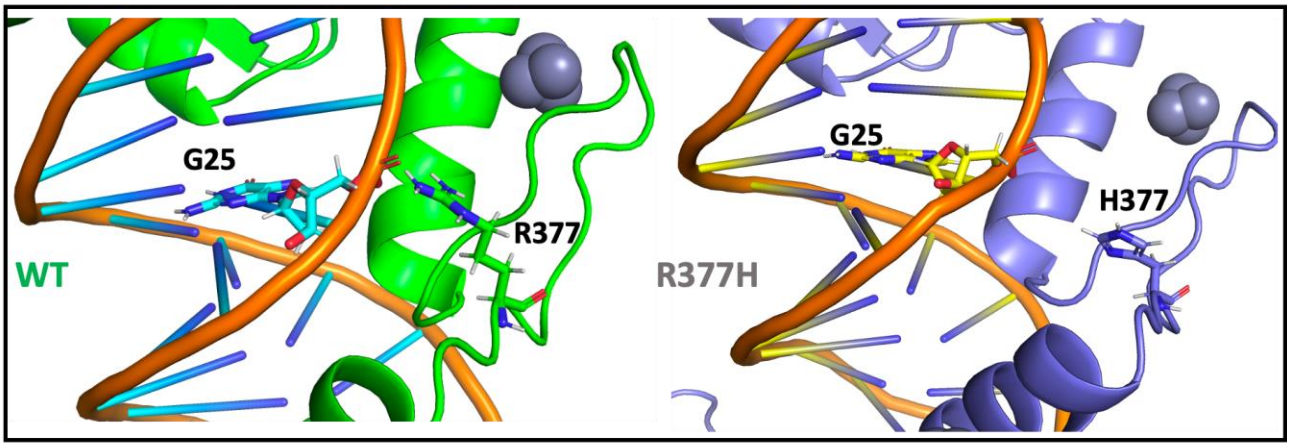

Supplementary Figure S3. WT and R377H proteins showing the position of the link region between ZF4 and ZF5 shifted away upon the R377H mutation.

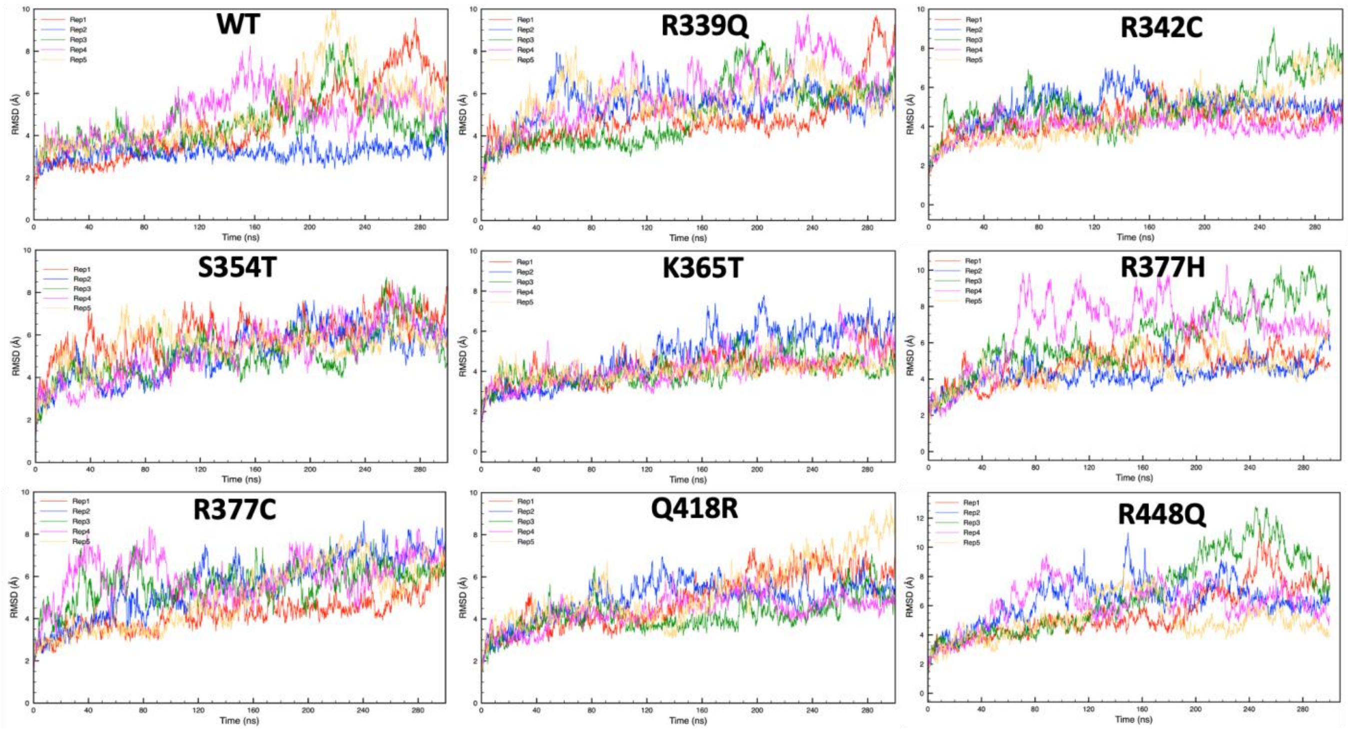

Supplementary Figure S4. Backbone root mean squared deviation across each trajectory for each simulated system.

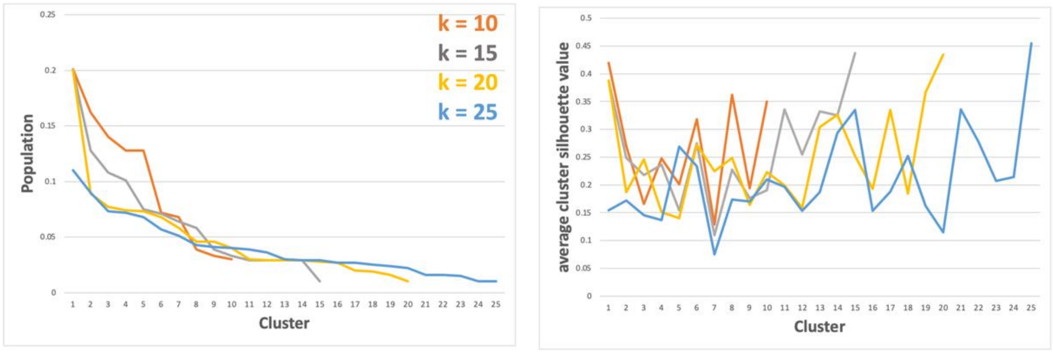

Number of Clusters	Average Silhouette Value
10	0.265
15	0.260
20	0.249
25	0.21

Supplementary Figure S5. Clustering analysis with increasing number of clusters. Population and average silhouette value.

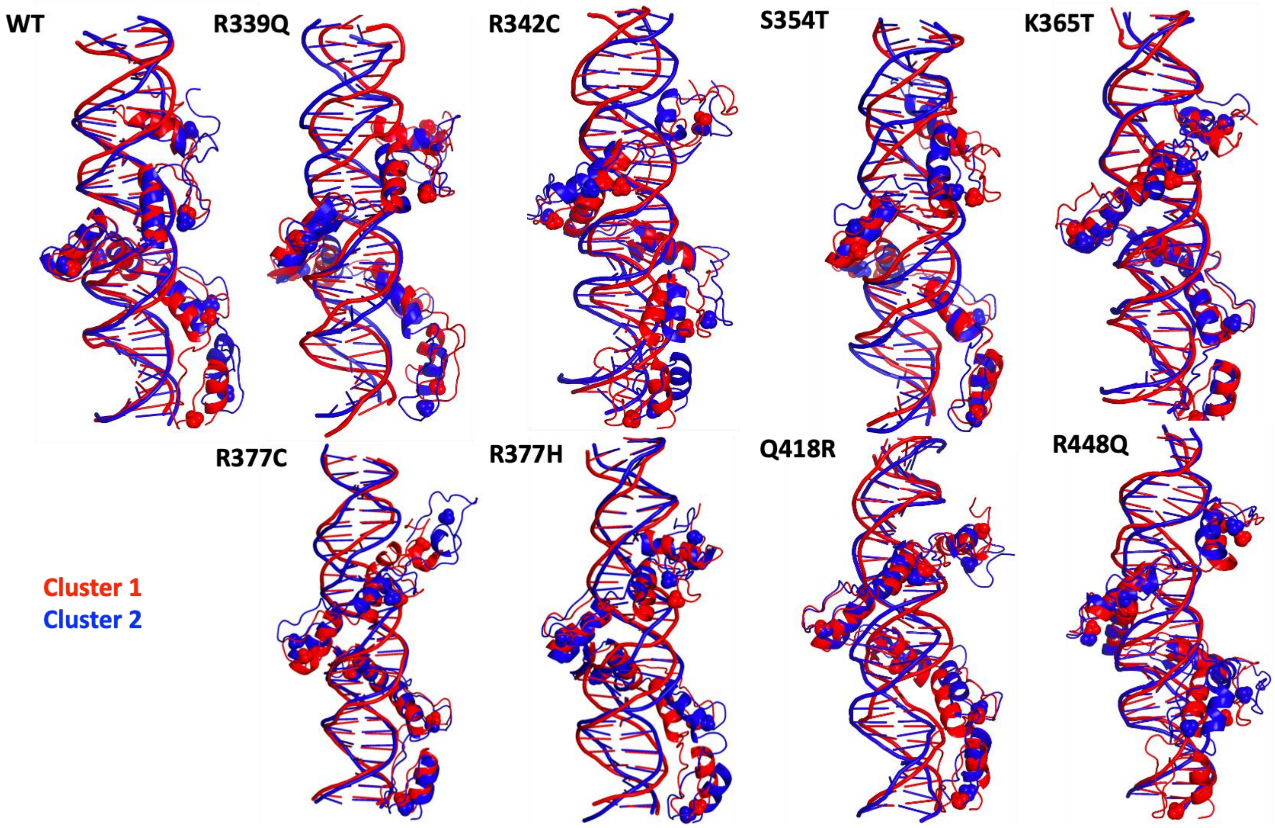

Supplementary Figure S6. Superposition of the representative structures of clusters 1 and 2 for each system.

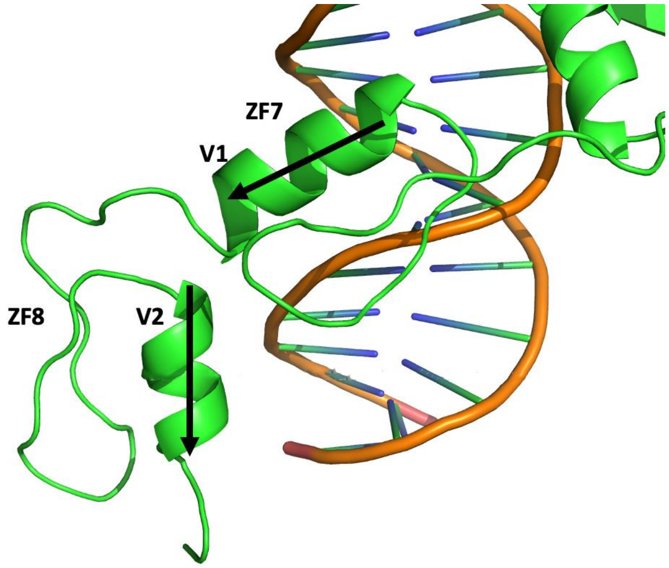

Supplementary Figure S7. Vectors defined in the α-helices of ZF7 and ZF8 used to calculate the angle formed between the two fingers.

2) Supplementary Tables

Supplementary Table S1. Secondary structure percentage of ZF3's α-helix.

	Total Secondary Structure	
Residue	WT	R342C
G335	0.9999	0.6505
E336	0.9956	0.4312
L337	0.9929	0.5263
V338	0.9996	0.9973
R339	1	1
H340	0.9714	0.9977
R341	1	1.0001
R342	1	1.0001
Y343	0.9999	1
K344	0.9999	1
Y345	0.9997	0.9973
K346	0.3008	0.2607
H347	0.1072	0.4617
T348	0.0263	0.2233

Supplementary Table S2. Cluster populations in fraction for each system simulated.

	**WT**	**R339Q**	**R342C**	**S354T**	**K365T**	**R377H**	**R377C**	**Q418R**	**R448Q**
**Cluster 1**	0.201	0.2	0.2	0.2	0.193	0.257	0.17	0.246	0.183
**Cluster 2**	0.162	0.2	0.199	0.2	0.146	0.117	0.158	0.123	0.173
**Cluster 3**	0.14	0.141	0.135	0.103	0.142	0.112	0.132	0.111	0.141
**Cluster 4**	0.128	0.107	0.101	0.102	0.109	0.098	0.128	0.104	0.127
**Cluster 5**	0.128	0.092	0.076	0.097	0.094	0.086	0.1	0.096	0.119
**Cluster 6**	0.072	0.075	0.07	0.079	0.091	0.086	0.099	0.085	0.073
**Cluster 7**	0.068	0.065	0.066	0.066	0.085	0.083	0.071	0.077	0.059
**Cluster 8**	0.039	0.059	0.065	0.06	0.069	0.059	0.066	0.068	0.05
**Cluster 9**	0.033	0.033	0.054	0.054	0.05	0.059	0.042	0.053	0.044
**Cluster 10**	0.03	0.029	0.034	0.039	0.021	0.043	0.034	0.036	0.031
